# Effectiveness and Safety of Biological Therapies in Very Severe Plaque Psoriasis: A Real-Life Retrospective Study

**DOI:** 10.3390/jpm14020186

**Published:** 2024-02-07

**Authors:** Giovanni Fiorillo, Luciano Ibba, Luigi Gargiulo, Alessandra Narcisi, Antonio Costanzo, Mario Valenti

**Affiliations:** 1 Department of Biomedical Sciences, Humanitas University, 20072 Pieve Emanuele, Italymario.valenti@hunimed.eu (M.V.); 2 Dermatology Unit, IRCCS Humanitas Research Hospital, 20089 Rozzano, Italy

**Keywords:** psoriasis, PASI, very severe psoriasis, biologics

## Abstract

Psoriasis can have a significant impact on quality of life and productivity, especially with increased severity. However, there is limited evidence on biologics’ efficacy in highly severe cases compared to moderate-to-severe ones. This study aimed to evaluate the effectiveness and safety of novel biological therapies in very severe psoriasis. We conducted a retrospective analysis on patients ≥ 18 years old affected by very severe psoriasis who had received a biological agent for at least 16 weeks. We used PASI to assess disease severity and effectiveness at weeks 16, 52, 104, and 156. Safety was evaluated by tracking treatment discontinuation rates and adverse events. This study included 29 males and 11 females, with a mean age of 55.80 years (SD 13.82). Cardiometabolic diseases were the most common comorbidities (25.00%). Twenty-eight (70.00%) patients had psoriasis involvement in at least one difficult-to-treat area. All patients completed 16 weeks of treatment. The mean PASI was 31.60 (SD 2.57) at baseline, 3.48 (SD 4.13) at week 16, 0.58 (SD 1.70) at week 52, 0.77 (SD 1.66) at week 104, and 1.29 (SD 2.12) at week 156. PASI90 and 100 were achieved by 52.50% and 30.00% of patients at week 16, by 96.15% and 80.77% at week 52, by 93.33% and 66.67% at week 104, and by 85.71% and 42.86% at week 156. PASIs ≤ 2 were achieved by 50.00% of patients at week 16, 88.46% at week 52, 86.67% at week 104, and 85.71% at week 156. Only two patients discontinued biologics due to complete remission, and mild AEs were reported by four patients. Our findings show that biologics are effective and well tolerated for treating very severe psoriasis, maintaining long-term effectiveness.

## 1. Introduction

Psoriasis is a chronic inflammatory disease affecting 2–4% of the general population worldwide [1,2]. Plaque psoriasis, the predominant variant, accounting for over 80% of cases, is characterized by erythematous scaly patches or plaques, typically appearing on extensor surfaces but also affecting intertriginous areas, palms, soles, and nails. Equally affecting men and women, it predominantly occurs in adults rather than children. Significant progress in understanding plaque psoriasis has been made in its pathogenesis, genetics, comorbidities, and biological treatment. Its pathogenesis involves a feed-forward mechanism of inflammation, notably through the T-helper cell type 17 pathway. Genetic factors play a crucial role, and environmental factors can exacerbate the condition. Other morphologic variants include guttate psoriasis, erythrodermic psoriasis, and pustular psoriasis [3].

Although, in most cases, the disease manifests on the skin, it is also associated with important comorbidities, including psoriatic arthritis, metabolic syndrome, cardiovascular diseases, inflammatory bowel diseases, type 2 diabetes mellitus, depression, and cancer [2,4,5,6,7]. The Psoriasis Area and Severity Index (PASI) is a tool used to evaluate and categorize the severity of psoriatic lesions and their response to treatment; it divides the body into four regions, the head, trunk, upper extremities, and lower extremities, and each of these areas is individually examined to determine the percentage of involvement, resulting in a numerical score ranging from 0 (no involvement) to 6 (90% to 100% involvement). Additionally, parameters such as erythema, induration, and scaling are assessed on a scale from 0 to 4. The PASI generates a numerical score ranging from 0 (no psoriasis) to 72; a score below 10 defines psoriasis as mild, 10 to 15 as moderate, and above 15 as severe [4,8]. With itching, pain, and scaling as its key symptoms, psoriasis can have an important impact on patient’s health-related quality of life (HRQoL) and work productivity, with growing real-world evidence showing an association of higher impairments with greater disease severity [9,10]. Conversely, reaching lower PASI values has been shown to correlate with better quality of life and work productivity [11].

The range of treatment options for managing psoriatic disease includes topical therapy, phototherapy, and systemic therapy [12]. The application of phototherapy in the treatment of moderate-to-severe psoriasis has declined with the introduction of biologics. Nonetheless, the primary forms of phototherapy for psoriasis include narrowband UV-B, broadband UV-B, and psoralen and UV-A (PUVA). Narrowband UV-B is generally preferred over broadband UV-B due to its higher efficacy, and it is also chosen over PUVA for its more favorable safety profile. Targeted phototherapy, like excimer light, is employed for localized psoriasis [3].

The selection of a particular treatment is predominantly influenced by the severity of the disease, the existence of comorbidities like psoriatic arthritis, and the patient’s treatment history, considering factors such as responsiveness and tolerability. The recognition that many psoriatic patients may be suitable candidates for systemic therapy has considerably expanded, and this is attributed, in part, to the significant progress made in our comprehension of psoriatic pathophysiology; it is now evident that psoriasis is not merely a skin condition but a chronic, systemic, immune-mediated disease, in which the skin plaques represent the visible dermatologic manifestation of this pathological process [12].

While mild forms can be managed by topical therapy, moderate-to-severe psoriasis typically requires systemic therapy. However, various barriers may block the initiation of systemic drugs, mainly due to the safety concerns shared by both patients and clinicians [4,12]. These concerns are justified, considering that traditional oral systemic therapies like methotrexate, cyclosporine, and acitretin come with potential organ toxicities, tolerability issues, and the need for regular laboratory monitoring [12,13].

Methotrexate is associated with common side effects such as fatigue, nausea, and diarrhea; it can cause congenital abnormalities or death in exposed fetuses, renal toxicity, myelosuppression, hepatotoxicity, pulmonary fibrosis, hemorrhagic enteritis, malignant lymphoma, severe skin reactions, and serious infections [12,13]. Although cyclosporine can rapidly improve psoriatic manifestations, its adverse events (e.g., hypertension, nephrotoxicity, severe infections, and increased risk of skin malignancies) and drug interactions limit its prolonged use [12,13]. Warnings and precautions associated with acitretin are birth defects in exposed fetuses, hepatotoxicity, hyperlipidemia, liver toxicity and toxic hepatitis, hyperostosis, pancreatitis, and pseudotumor cerebri (benign intracranial hypertension); women of childbearing potential must not become pregnant during treatment and for at least three years after discontinuing treatment [12,14].

Biologics are monoclonal antibodies targeting cytokines such as interleukin(IL)-17, IL-23, IL-12/23, and tumor necrosis factor-alpha (TNF-α); due to their great efficacy and tolerance, they are increasingly used for treating moderate-to-severe psoriasis when the patient does not tolerate the traditional systemic therapies due to adverse effects or inefficacy [4,13]. The great efficacy of biologics drives higher expectations and goals in the management of moderate-to-severe psoriasis; a ≥90% improvement in PASI (PASI90) or the achievement of absolute PASI ≤ 2 until 16 weeks of treatment are the current aims of treatment [2,15]. Yet, there is little evidence from clinical trials and observational open-label studies that highly severe psoriasis responds to biologics with the same effectiveness and rapidity as moderate-to-severe psoriasis.

Newer biological treatments have been associated with a reduced risk of several psoriatic comorbidities (e.g., type 2 diabetes mellitus, myocardial infarction, hypertension, and hyperlipidemia) and seem to improve the metabolic profile, leading from a pro-inflammatory to an anti-inflammatory phenotype [13,16,17]. In addition, they have demonstrated safety in special populations such as immunocompromised patients, cancer patients, and women with childbearing potential [1,2,18]. However, despite nearly two decades of biologics use in psoriatic disease, some dermatologists still harbor reservations regarding their long-term safety [12].

While biological agents are generally considered safe and well tolerated, like any medication, they carry potential adverse effects related to their mechanism of action, dosage, or other factors. Patients receiving TNF inhibitors have reported increased rates of upper respiratory tract infections, pharyngitis, sinusitis, and rhinitis; moreover, cases of tuberculosis have emerged during clinical trials of TNF inhibitors. Long-term safety studies of anti-IL-12/23 agents have identified upper respiratory tract infections, nasopharyngitis, headache, and arthralgia as the most common adverse events (AEs). Psoriatic patients treated with IL-17 inhibitors present an increased risk of Candida infection, and these medications may exacerbate or even induce inflammatory bowel disease (IBD) [19]. It is crucial to have more evidence regarding both the efficacy and safety of existing treatments in different subgroups of psoriatic patients to make informed therapeutic decisions [19].

On these premises, this study aimed to evaluate the effectiveness and safety of novel biological therapies in patients affected by very severe psoriasis.

## 2. Materials and Methods

We conducted a retrospective medical record analysis on patients ≥ 18 years old affected by very severe psoriasis (i.e., PASI ≥ 30) receiving a biological agent approved for psoriasis for at least 16 weeks at the Humanitas Research Hospital, Milan, Italy. We used the Italian adaptation of the EuroGuiDerm guidelines on the systemic treatment of chronic plaque psoriasis [20] to evaluate patient eligibility for biological therapy. The following information was retrieved from medical records: sex, age, body mass index (BMI), year of psoriasis onset, comorbidities (i.e., psoriatic arthritis, cardiovascular diseases, arterial hypertension, type 2 diabetes mellitus, hypercholesterolemia, viral hepatitis B or C, latent tuberculous infection, and personal history of malignancies), involvement of difficult-to-treat areas, current and previous biological treatments for psoriasis, causes of discontinuation, and AEs during treatment with biological agents.

We used PASI to assess disease severity at weeks 16, 52, 104, and 156 after starting biological therapy. Effectiveness was evaluated by registering, at weeks 16, 52, 104, and 156 the percentages of patients achieving an improvement of 90% and 100% in PASI (PASI90 and PASI100, respectively) and the percentage of patients who reached PASI ≤ 2. Safety was assessed by evaluating the rates of treatment discontinuation and the occurrence of AEs at each time point.

Stata/SE 17.0 software was used for statistical analysis, and Microsoft Excel (version 2401)was used to generate tables and figures. Quantitative data are expressed as mean ± standard deviation (SD), and qualitative data as absolute frequency and percentages. The chi-square and Exact Fisher’s tests were used to analyze differences in PASI90, PASI100, and PASI ≤ 2 responses between the anti-IL-17, anti-IL-23, and anti-IL-12/23 subgroups. Patients who achieved complete remission of psoriasis were included in the statistical analysis. Data of patients who missed the scheduled visits were analyzed by using the observed case (OC) approach. A *p*-value of <0.05 was considered statistically significant.

Since the study protocol did not diverge from standard clinical practice, ethical approval was not required by the committee of our institution. Written informed consent for the retrospective study of the gathered data was obtained from all enrolled patients during routine clinical practice.

## 3. Results

This study included 40 patients, 29 (72.50%) males and 11 (27.50%) females. The mean age was 55.80 years (SD 13.82). The mean BMI was 29.27 kg/m^2^ (SD 10.19); as data from 20 patients were missing, BMI was calculated in 20 (50.00%) patients. Cardiometabolic diseases (i.e., cardiovascular diseases, arterial hypertension, type 2 diabetes mellitus, and hypercholesterolemia) were the most common comorbidities (10, 25.00%), followed by psoriatic arthritis (8, 20.00%), obesity (5, 12.50%), personal history of malignancies (1, 2.50%), and viral hepatitis B (1, 2.50%). The mean disease duration was 20.33 years (SD 15.27). Twenty-eight (70.00%) patients had psoriasis involvement in at least one difficult-to-treat area (i.e., the scalp, face, nails, genitals, and palmoplantar regions), and 33 (82.50%) were bio-naïve. The most prescribed biologic was risankizumab (21 patients, 52.50%), followed by ixekizumab (5, 12.50%), guselkumab (4, 10.00%), and ustekinumab (4, 10.00%). All the main characteristics of our population at baseline are shown in Table 1.

All 40 patients completed 16 weeks of biological treatment, whereas 26 (65.00%) reached 52 weeks, 15 (37.50%) 104 weeks, and 15 (37.50%) 156 weeks.

**Figure 1:**
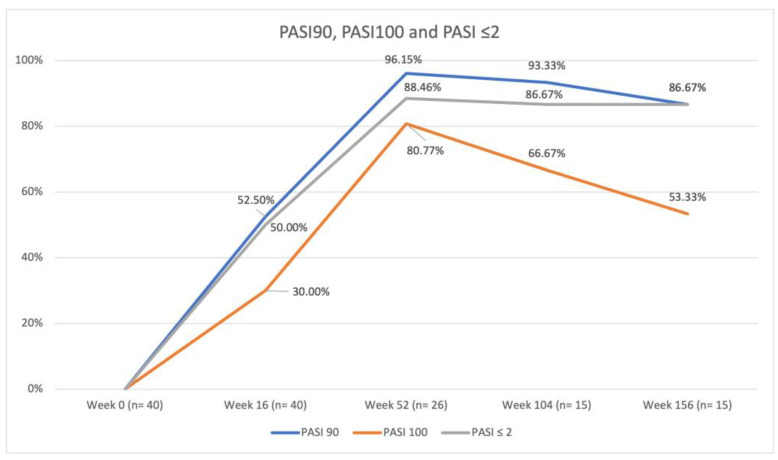
Percentage of patients achieving PASI90, PASI100, and absolute PASI ≤ 2 compared with baseline PASI. Analysis was performed on 40 patients at weeks 0 and 16, on 26 patients at week 52, and on 15 patients at weeks 104 and 156. Abbreviations: PASI, Psoriasis Area and Severity Index.

The results concerning the effectiveness profile are summarized in Figure 1.

The mean PASI was 31.60 (SD 2.57) at baseline, 3.48 (SD 4.13) at week 16, 0.58 (SD 1.70) at week 52, 0.77 (SD 1.66) at week 104, and 1.29 (SD 2.12) at week 156. PASI90 and 100 were achieved by 52.50% and 30.00% of patients at week 16, by 96.15% and 80.77% at week 52, by 93.33% and 66.67% at week 104, and by 86.67% and 53.33% at week 156, respectively. PASIs ≤ 2 were reached by 50.00% of patients at week 16, 88.46% at week 52, 86.67% at week 104, and 86.67% at week 156. The differences in PASI90, PASI100, and PASI ≤ 2 responses between the anti-IL-17, anti-IL-23, and anti-IL-12/23 subgroups were not statistically significant.

Biological treatment was discontinued in two (5.00%) patients treated with risankizumab due to complete remission. AEs were reported by four (10.00%) patients, of which three had upper respiratory tract infections and one had oral candidiasis. No severe AEs or new safety findings were detected during biological treatment. The patient affected by viral hepatitis B did not develop disease reactivation. No new malignancies and no recurrence or progression of previous cancer were found in our cohort.

## 4. Discussion

Plaque psoriasis, constituting around 80% to 90% of all psoriasis cases, manifests as well-defined, erythematous, and scaly patches or plaques. While it can appear anywhere on the body, common sites include the scalp, trunk, gluteal fold, and extensor surfaces like elbows and knees. Lesions vary from small erythematous papules to large, thick plaques, often well demarcated and symmetrical [3]. The impact on patients’ functional, psychological, and social outcomes is highly influenced by the body area where the disease occurs, especially when plaques involve sensitive areas like the face, palms, soles, nails, skin folds (inverse psoriasis), and genital region (seen in about one-third of patients). When the palms and soles are affected, thick, scaly, and painful plaques limit hand and foot function; nail involvement may lead to pitting, onycholysis, and nail dystrophy, leading to functional and esthetic damage [3,9,10].

Extensive real-world evidence indicates that higher psoriasis severity is linked to lower HRQoL and increased impairments in work productivity [9,10]. This is also explained by the higher prevalence of comorbidities compared to the general population; severe psoriasis is associated with a significant increased risk of myocardial infarction, coronary artery disease, stroke, and cardiovascular mortality, as well as metabolic syndrome, type 2 diabetes mellitus, inflammatory bowel diseases, depression, anxiety, and suicidal ideation [2,3,4,5,6,7].

Since clinical improvement aligns with a notable enhancement in quality of life, effective treatment is essential for patients with very severe psoriasis who are either not controlled by, intolerant to, or have contraindications to currently available systemic therapies [9]. In the last few years, evidence regarding the effectiveness of different biological drugs for psoriasis in a real-life setting has increased, whereas analyses of treatment survival are still limited to registry studies, multicenter studies, or small single-center case series [2,4,21]. Nevertheless, clinical trials and observational studies provide scant proof that biologics yield a response in highly severe psoriasis comparable to that observed in moderate-to-severe psoriasis.

While the prevalence of psoriasis among men and women is estimated to be similar [5,22], we found an imbalance in sex proportions (29 males, 11 females) in our population. This could be explained by the selection of patients with very severe psoriasis, as the severity of the disease can vary according to sex. Male patients tend to have higher PASI values compared to women, and several studies have shown that men are more likely to receive systemic therapies; the reason for this has been assumed to be the difference in disease severity [5,22,23].

Our study highlights that biologics were an effective treatment for patients affected by very severe psoriasis, with 96.15% achieving PASI90 and 88.46% reaching an absolute PASI ≤ 2 after 52 weeks of therapy. The lower PASI90 and PASI100 responses after 16 weeks of treatment are probably ascribed to the high baseline PASI (≥30) and percentage of patients with psoriasis involvement of at least one difficult-to-treat area (70.00%), which are known to have a slower response to biologics [1,24,25]. Interestingly, the line chart for PASI90, PASI100, and PASI ≤ 2 shows an inflection point at week 52 (Figure 1). Some patients lose the achieved response over time, and this has predominantly been linked to low circulating drug levels [26,27,28,29]. Indeed, growing evidence suggests that therapeutic drug monitoring (TDM), encompassing the assessment of trough concentrations and anti-drug antibodies in conjunction with clinical response, is emerging as a significant tool for clinical decision-making; this includes applications such as monitoring patient adherence and managing patients who experience a decline in response over time [30,31]. Biological therapies are generally effective in achieving the clearance of psoriasis in most cases. Nevertheless, given the chronic nature of this disease, patients need to maintain a response to treatment [30]. A real-world study conducted by McLean et al. [30] revealed that approximately 50% of psoriatic patients, who initially achieved near-complete skin clearance after 6 months of receiving a biologic, experienced a decline in this level of response over a 24-month follow-up period. Multiple factors, including differences in baseline patient characteristics, such as mean body mass index, race, and ethnicity, may influence the response to treatment [31,32]. Loss of efficacy represents the primary reason for patients withdrawing from biological therapy, with about 47% of patients discontinuing treatment after 3 years [28,33]. A greater risk of loss of response has been associated with increased Dermatology Life Quality Index (DLQI) and more burdensome disease symptoms (including fatigue); this emphasizes the correlation between treatment effectiveness and enhanced patient satisfaction or quality of life [30]. Understanding the factors influencing the durability (maintenance of response over time) of near-complete skin clearance in patients treated with biologics should be an important endpoint for future studies since most evidence has come primarily from randomized clinical trials, which may not fully reflect the real-world experience of psoriatic patients [34,35].

Regarding safety, we only observed mild AEs, and there was no discontinuation due to drug toxicities in our cohort. Long-term observational data consistently demonstrate a reassuring safety profile of newer immunomodulatory agents for psoriasis. Nonetheless, the handling of AEs remains a key factor in treatment-related decision-making, as attributes like “overall safety” and “low potential for AEs” were rated highest in real-world use when considering treatments for moderate-to-severe psoriasis. In the short term, anti-IL-23 agents (guselkumab, risankizumab, and tildrakizumab) generally exhibited lower rates of any AE compared to anti-IL-17 agents (brodalumab, secukinumab, and ixekizumab), whereas there is limited evidence in the literature regarding comparisons of long-term safety outcomes, but to date, risankizumab has emerged with the most favorable benefit–risk profile when compared to anti-IL-17 agents, adalimumab, and ustekinumab [19]. This difference may be attributed to the distinct mechanisms of action between anti-IL-23 and anti-IL-17 agents. IL-23 primarily contributes to protection against bacterial and parasitic infections, while IL-17 is involved in host defense against various infections [19]. Both human and mouse studies have shown the involvement of IL-17 in mucocutaneous defense against Candida albicans. Real-world observational studies have uncovered significant associations between IL-17 inhibitors and occurrences of oropharyngeal and esophageal candidiasis; specifically, the associated risks were found to be four- to ten-fold higher compared to those associated with TNF-α inhibitors, while the risk of oral and gastrointestinal candidiasis in psoriatic patients using IL-23 inhibitors remains uncertain and requires assessment in real-life settings [36]. Due to the inhibition of crucial cytokines in innate and adaptive immunity by biological therapies, concerns have arisen about the development of opportunistic infections, including the activation of tuberculosis and herpes zoster. The host defense mechanism against Mycobacterium tuberculosis involves Th1 cells and cytokines like IFN-γ, IL-12, and TNF-α. TNF-α inhibitors, which suppress Th1 responses, have been associated with an increased risk of tuberculosis activation since their introduction in treating rheumatoid arthritis and ankylosing spondylitis. Therefore, it is strongly recommended to conduct screening tests (interferon-γ release assay and chest X-ray) and annual screenings for latent tuberculosis. For patients with latent tuberculosis, 6- to 9-month chemoprophylaxis with isoniazid or rifampicin monotherapy is recommended, starting 3 weeks before the initiation of biological treatment [36]. The role of Th17 cells in the host defense against Mycobacterium tuberculosis remains a subject of controversy. While preclinical studies indicate that IL-17 induces neutrophil recruitment and a local inflammatory response through cytokine and chemokine secretion, the functions of IL-23 and IL-17 in tuberculosis seem more nuanced compared to Th1 cytokines. In contrast to the numerous cases of tuberculosis reactivation during TNF-α inhibitor treatment and a few cases linked to IL-12/23 inhibitors, there have been no reported instances in patients exposed to IL-23 or IL-17 inhibitors in both clinical and real-world settings, aligning with our findings [36]. Some authors, based on these clinical data, express skepticism regarding annual screening for asymptomatic patients receiving IL-23 and IL-17 inhibitors [36,37]. The risk of opportunistic infection by the varicella-zoster virus increases in psoriatic patients undergoing biological therapy. Recent population-based observational research indicated a lower risk of herpes zoster associated with IL-17 and IL-23 inhibitors compared to TNF-α inhibitors [36,38,39].

As mentioned before, psoriasis also poses a risk for myocardial infarction, coronary artery disease, and stroke, particularly in younger patients or those with severe disease [36,40,41]. The potential role of IL-17 in cardiovascular disease is suggested by the elevated mortality and recurrent acute myocardial infarction in patients with low serum IL-17 levels [36]. However, the beneficial impact of IL-17 blockade on cardiovascular disease risk in psoriatic patients remains uncertain. Evaluations using coronary computed tomography and angiography in moderate-to-severe psoriasis patients demonstrated that biological therapies, including IL-17 inhibitors, were associated with a reduction in high-risk coronary plaque phenotypes. Conversely, low plasma IL-17A levels were linked to an increased incidence of cardiovascular diseases in patients with moderate-to-severe psoriasis [36,42]. However, treatment with secukinumab did not show a beneficial effect on aortic vascular inflammation, an imaging biomarker of cardiovascular disease risk, and clinical trials did not reveal a significant association between IL-17A blockade and major adverse cardiovascular events (MACEs) [36,43,44].

In addition, current guidelines advise against administering IL-17 inhibitors to psoriatic patients with a personal history of or active IBD (i.e., ulcerative colitis and Crohn’s disease). Individuals with psoriasis, particularly the young and those with severe psoriasis, face an elevated risk of IBD. Nevertheless, the potential association between IL-17 blockade and the onset of IBD in psoriatic patients, especially in young individuals with yet-to-manifest IBD symptoms, remains uncertain [36].

In conclusion, biologics have significantly improved the treatment of psoriasis, and now, they represent the standard treatment for moderate-to-severe psoriasis, especially in patients who do not respond to conventional systemic treatments, such as methotrexate, cyclosporin, or acitretin [4]. Confirming their effectiveness and safety in very severe psoriasis could be an interesting new goal, as increased psoriasis severity has been associated with worsened patient-reported outcomes (pain, itching, fatigue; DLQI; EuroQoL Visual Analogue Scale [EQ-VAS]; Work Productivity and Activity Impairment [WPAI]) [10]. Moreover, there are no current international guidelines that specify first-choice biologics for severe psoriasis [4]. The addition of the “very severe” category may shed light on specific unmet medical needs in this segment of the population with psoriasis [10]. Our experience adds new evidence on the effectiveness and tolerability of biological therapies in patients affected by very severe psoriasis. However, the limitations of our study (including its retrospective nature and the small sample) do not allow us to compare the different classes of biologics in these populations. Further studies are needed to fully understand how differences in PASI categorization may impact the first-choice biological agent in patients affected by very severe psoriasis.

## Figures and Tables

**Table 1:** Clinical and demographic characteristics of our population at baseline.

Number of Patients	40
Male, *n* (%)	29 (72.50)
Age (years), mean ± SD *	55.80 ± 13.82
Disease duration (years), mean ± SD	20.33 ± 15.27
BMI **, mean ± SD	29.27 ± 10.19
Obese, *n* (%)	5 (12.50)
Cardiometabolic comorbidities, *n* (%)	10 (25.00)
Personal history of malignancies, *n* (%)	1 (2.50)
Latent tuberculosis infection, *n* (%)	0 (0.00)
Hepatitis C, *n* (%)	0 (0.00)
Hepatitis B, *n* (%)	1 (2.50)
PsA ***, *n* (%)	8 (20.00)
≥1 Difficult-to-treat areas, *n* (%)	28 (70.00)
PASI **** baseline, mean ± SD	31.60 ± 2.57
Bio-naïve, *n* (%)	33 (82.50)
Anti-IL *****-23 agents	
Risankizumab, *n* (%)	21 (52.50)
Guselkumab, *n* (%)	4 (10.00)
Tildrakizumab, *n* (%)	1 (2.50)
Anti-IL-17 agents	
Ixekizumab, *n* (%)	5 (12.50)
Secukinumab, *n* (%)	2 (5.00)
Brodalumab, *n* (%)	2 (5.00)
Bimekizumab, *n* (%)	1 (2.50)
Anti-IL-12/23 agents	
Ustekinumab, *n* (%)	4 (10.00)

Note: * standard deviation; ** body mass index; *** psoriatic arthritis; **** Psoriasis Area and Severity Index; ***** interleukin.

## Data Availability

Data are available on request from the authors.
